# Safeguarding marine life: conservation of biodiversity and ecosystems

**DOI:** 10.1007/s11160-022-09700-3

**Published:** 2022-03-07

**Authors:** Delphi Ward, Jessica Melbourne-Thomas, Gretta T. Pecl, Karen Evans, Madeline Green, Phillipa C. McCormack, Camilla Novaglio, Rowan Trebilco, Narissa Bax, Madeleine J. Brasier, Emma L. Cavan, Graham Edgar, Heather L. Hunt, Jan Jansen, Russ Jones, Mary-Anne Lea, Reuben Makomere, Chris Mull, Jayson M. Semmens, Janette Shaw, Dugald Tinch, Tatiana J. van Steveninck, Cayne Layton

**Affiliations:** 1 Institute for Marine and Antarctic Studies, University of Tasmania, Castray Esplanade, Hobart, TAS 7001 Australia; 2 Centre for Marine Socio-Ecology, University of Tasmania, Hobart, TAS 7001 Australia; 3 CSIRO Oceans and Atmosphere, Castray Esplanade, Hobart, TAS 7001 Australia; 4 Adelaide Law School, The University of Adelaide, North Terrace, Adelaide, SA 5005 Australia; 5 South Atlantic Environmental Research Institute, Stanley, Falkland Islands; 6 Silwood Park Campus, Department of Life Sciences, Imperial College London, Berkshire, SL5 7PY UK; 7 Department of Biological Sciences, University of New Brunswick, PO Box 5050, Saint John,, New Brunswick E2L 4L5 Canada; 8 Hereditary Chief, Haida Nation, PO Box 1451, Skidegate, B.C. V0T 1S1 Canada; 9 Faculty of Law, University of Tasmania, Hobart, TAS 7001 Australia; 10 Integrated Fisheries Lab, Department of Biology, Dalhousie University, Halifax, NS B3H 4R2 Canada; 11 Tasmanian School of Business & Economics, University of Tasmania, Hobart, TAS 7001 Australia; 12 Carmabi, Caribbean Research and Management of Biodiversity, Piscaderabaai z/n, Willemstad, Curaçao

**Keywords:** Ecosystem management, Ecosystem services, Indigenous knowledge, Integrated management, Stewardship, Sustainable Development Goals, Foresighting/hindcasting

## Abstract

Marine ecosystems and their associated biodiversity sustain life on Earth and hold intrinsic value. Critical marine ecosystem services include maintenance of global oxygen and carbon cycles, production of food and energy, and sustenance of human wellbeing. However marine ecosystems are swiftly being degraded due to the unsustainable use of marine environments and a rapidly changing climate. The fundamental challenge for the future is therefore to safeguard marine ecosystem biodiversity, function, and adaptive capacity whilst continuing to provide vital resources for the global population. Here, we use foresighting/hindcasting to consider two plausible futures towards 2030: a business-as-usual trajectory (i.e. continuation of current trends), and a more sustainable but technically achievable future in line with the UN Sustainable Development Goals. We identify key drivers that differentiate these alternative futures and use these to develop an action pathway towards the desirable, more sustainable future. Key to achieving the more sustainable future will be establishing integrative (i.e. across jurisdictions and sectors), adaptive management that supports equitable and sustainable stewardship of marine environments. Conserving marine ecosystems will require recalibrating our social, financial, and industrial relationships with the marine environment. While a sustainable future requires long-term planning and commitment beyond 2030, immediate action is needed to avoid tipping points and avert trajectories of ecosystem decline. By acting now to optimise management and protection of marine ecosystems, building upon existing technologies, and conserving the remaining biodiversity, we can create the best opportunity for a sustainable future in 2030 and beyond.

## Introduction

The diversity of life in the oceans, marine biodiversity, is declining globally at an alarming rate (Lotze et al. 2019; Worm et al. 2006), driven by multiple interacting anthropogenic stressors, which are degrading marine ecosystem function, shifting species’ distributions, and initiating the formation of novel ecosystems with unknown characteristics and services (e.g. Harborne and Mumby 2011; Pecl et al. 2017). These losses threaten the wellbeing and survival of much (arguably all) of humankind that fundamentally depends on the many services provided by marine biodiversity and ecosystems, including climate regulation, coastal protection, food and medicinal products, recreational activities, and livelihoods (Peterson and Lubchenco 1997; Selig et al. 2018). These ecosystems also possess unique, often intangible, inherent values making them crucial to the health and wellbeing of peoples around the world. As such, safeguarding marine biodiversity and ecosystem function into the future is a task of critical importance. The challenge is to conserve existing biodiversity, while increasing the capacity to forecast ecological trajectories and future ecosystem states to inform sustainable management long-term (Cheung 2019). Ecological forecasts are needed for developing adaptation strategies to guide ecosystems towards states that support a high diversity of functions and species. Stemming the rate of biodiversity loss at all levels – including genetic, taxonomic, community, ecosystem, and functional diversity – will leave marine species and ecosystems with a wider breadth of adaptive pathways, thus increasing the likelihood of resilience, rather than extinction, in future seas.

Marine ecosystems and biodiversity have undergone rapid and profound changes in the Anthropocene (e.g. Estes et al. 2011; Jackson 2001; Pimiento et al. 2020). Marine and coastal ecosystem changes resulting from human activity have steeply accelerated in the last ~ 150 years (Bindoff et al. 2019; Halpern et al. 2019). Identifying pre-industrial environmental ‘baselines’ to enable the quantification of ecological changes is challenging and often unfeasible, not only because ecosystems continuously change in response to environmental phenomena, but also since in many cases anthropogenic pressures began before Western scientific monitoring commenced (Jackson 1997; Jennings and Blanchard 2004; Roberts 2007). An emerging “mass extinction” event is thought to be underway in the oceans (Lotze et al. 2019; Payne et al. 2016) caused by the combined (and sometimes synergistic) effects of overfishing (Blanchard et al. 2017; FAO 2018), habitat degradation and loss (IPBES 2019), pollution, eutrophication, oxygen depletion, introduced pests, and ocean warming (Breitburg et al. 2018; Doney 2010). These cumulative stressors have, in some cases, led to dramatic and difficult-to-reverse shifts in ecosystem state – or “ecosystem collapses” (e.g. Beaugrand et al. 2015; Biggs et al. 2018; Möllmann and Diekmann 2012). Indeed, historical ecosystem states may have increasingly limited relevance in the context of substantial and ongoing impacts, particularly as a result of climate change. Despite these pervasive impacts and trajectories of ecosystem degradation, there is still reason for hope, as marine biodiversity and ecosystems continue to support the services upon which societies rely and the recovery of many degraded marine ecosystems is considered achievable by 2050, if there is sufficient will and targeted effort (Duarte et al. 2020).

A common approach to conservation in the marine realm is the implementation of ‘Marine Protected Areas’ (MPAs) that secure ecosystems by separating them from human use and/or limiting extractive/destructive processes. This approach is upheld in United Nations processes including the Aichi Targets of the Convention on Biological Diversity, and the 2030 Agenda and Sustainable Development Goals (SDGs). While MPAs are, and will continue to be, a fundamental and effective conservation tool when properly implemented and managed (see Edgar et al. 2014; Gownaris et al. 2019), human population growth, and activities contributing to unsustainable lifestyles, continue to threaten marine ecosystems beyond the boundaries of MPAs (Cafaro 2021; Halpern et al. 2019). Safeguarding marine biodiversity and ecosystems into the future will therefore require more holistic and inclusive approaches. It is not possible to secure all (or even the majority) of the marine estate as MPAs, nor is it desirable in contexts where stewardship is high and people are able to live in balance with ecosystems (Cinner et al. 2016; Gilchrist et al. 2020; Stewart et al. 2020). Indeed, some evidence suggests that the greatest conservation outcomes arise where communities are most intimately connected to their local ecosystems and the associated decision-making processes (e.g. Nikitine et al. 2018; Wells and White 1995). It is therefore imperative that we consider how to improve and optimise conservation outcomes in ‘non-protected’ areas. This will require a fundamental recalibration of the way individuals, communities, industries, and financial markets perceive and interact with the marine environment. Setting ambitious goals for marine conservation is fundamental (Díaz et al. 2020), but importantly, failure to achieve previous globally agreed biodiversity conservation targets (Díaz et al. 2019; UN 2020) highlights the need to innovate our approach to achieving conservation goals.

Here, we use a forecasting/hindcasting approach to consider two plausible futures for 2030. These two futures encompass 1) a business-as-usual future that results from a continuation of current trajectories, and 2) a more sustainable, aspirational, but technically achievable future in line with progress towards achieving the UN SDGs. The coming decade will be defined by great uncertainty and complexity, with major transformations needed to move towards a sustainable future (Sachs et al. 2019). Development and communication of a ‘mobilising narrative’ that envisions a positive yet possible future is a first step towards outlining concrete actions to anticipate and constructively respond to future challenges (Nash et al. 2021a, this issue). We acknowledge that the current COVID-19 pandemic is causing major changes to economies and socio-ecological systems at local, national and global scales. The business-as-usual scenario we describe here is based on evidence from the recent past prior to the pandemic, and assumes a general return to this trajectory over the next few years. We note however, that current disruptions to the global ocean, environment, and society because of COVID-19 may present a platform for change and an opportunity to ‘reset’ trajectories in the coming decade (Sandbrook et al. 2020). The sustainable future presented here is one option for such a shift. Our goal is to highlight potential opportunities associated with moving towards one version of a more-sustainable future, rather than providing an exhaustive exploration of every option.

The UN Decade of Ocean Science for Sustainable Development (2021–2030) is a timely opportunity to align global focus on arresting and reversing the degradation of marine environments, and to ensure ocean science supports improvements towards the sustainable and equitable development of the world’s oceans (Pendleton et al. 2020). In considering our two plausible futures for 2030, we identify key drivers of change that differentiate these futures, and use these as a basis for identifying concrete actions that align with achieving the more sustainable future. We identify choices and actions across various scales (e.g. local, regional, national, international) to arrive at a more desirable future for the oceans in the context of our rapidly changing climate. The aspirational, more sustainable, scenario is intended to highlight a vision of what is achievable if society “chooses” to work collaboratively towards a future more closely aligned with achieving the UN SDGs (Nash et al. 2021a, this issue, for additional context).

## Methods

This paper is part of the larger 'Future Seas' project, the aim of which was to leverage interdisciplinary knowledge to address the grand challenges for the oceans in the coming decade. As part of Future Seas, the approach for addressing these grand challenges was developed by a core team (Nash et al. 2021a) and discussed, tested and refined through a series of workshops with the broader group of Future Seas participants. Future Seas participants were assembled into author teams, and each team addressed a separate grand challenge following the same methods, which are described in detail by Nash et al. (2021a) and summarised here.

The overarching goal of this paper was to describe a technically feasible pathway towards 2030 through which we could improve the status of marine ecosystems and biodiversity globally (or at least, stem their loss). In this process, subgoals included 1) identifying 4–6 key drivers of change in marine ecosystems and biodiversity; 2) describing the likely business-as-usual future for 2030 based on current trends in these drivers; 3) describing a more sustainable but achievable future state of the drivers and human-marine ecosystem interactions; 4) identifying specific actions that could feasibly shift us from the business-as-usual trajectory towards the more sustainable future we described; 5) identifying timeframes, key actors and scale for actions in the pathway.

**Fig. 1:**
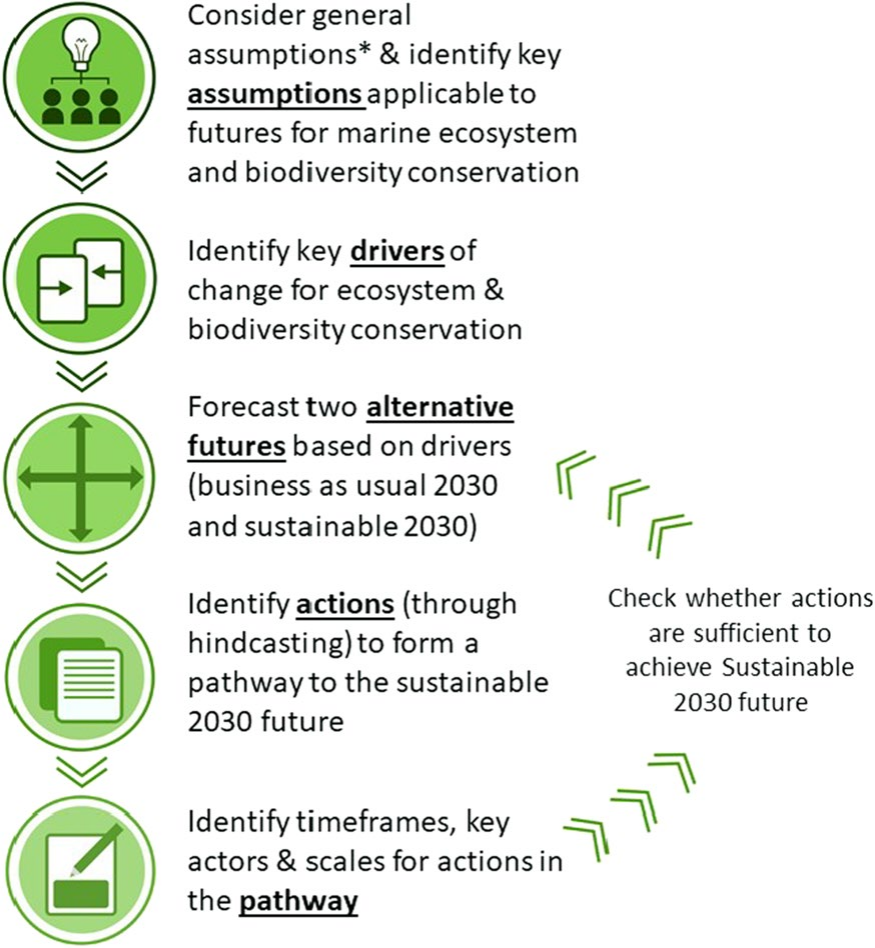
An overview of the methods followed to develop alternative scenarios of 2030 for marine ecosystem and biodiversity conservation (* from Nash et al. 2021a, this issue)

Our approach for developing these alternative futures and pathway was to apply established foresighting and hindcasting techniques that are used in futures analysis and scenario development in the socio-ecological literature (Nash et al. 2021a; Planque et al. 2019; Rintoul et al. 2018) (also see Fig. 1 for an overview). The process involved collaboration among our interdisciplinary co-author team for co-constructed scenario development during a series of workshops and meetings. Disciplines represented by our team include law, governance, management, fisheries, and economics, along with Indigenous leadership, ecologists and other biophysical scientists. Given our location, most authors are Australian (12), but authors also come from UK (3), Canada (2), Haida Nation (Canada, 1), New Zealand (1), Italy (1), Germany (1), The Netherlands (1) and Kenya (1). The team also consulted with an international group of Traditional Owners and Indigenous knowledge holders, and community representatives (see Fischer et al. 2021; Mustonen et al. 2021, both this issue).

Prior to developing future scenarios, we considered the underlying assumptions articulated in Nash et al. (2021a) as being broadly applicable across a wide range of global challenges for marine systems and confirmed their relevance to developing the two plausible futures for marine biodiversity and conservation by 2030. Assumptions included i) general ocean resource use and knowledge production continue, ii) no new major international agreements are ratified (however, existing discussions will continue), iii) the globe is locked into some degree of climate change over the coming decade, iv) human populations will continue to increase and v) no new large-scale human conflicts emerge. Moreover, we assumed that vi) demand for seafood will continue to rise and that vii) food insecurity, in terms of availability, access, utilisation and stability, will remain a challenge for some regions and people (see Farmery et al. 2021, this issue), and that viii) climate-driven redistribution of species in the ocean will continue as per projected trends (see Melbourne-Thomas et al. 2021, this issue).

To identify broad drivers of change relevant to the state of marine ecosystem and biodiversity, we first brainstormed all drivers affecting marine ecosystems, with participants writing individual drivers on post-it notes. In doing so, we aimed to identify Political, Economic, Social, Technological, Legal and Environmental (PESTLE) drivers to ensure consideration of different driver types (Nash et al. 2021a). We then grouped these individual drivers into broader, umbrella drivers. For example, fishing-related drivers, deep-sea mining, shipping, marine renewable energy were all eventually grouped together under the sectoral stewardship umbrella driver. These umbrella drivers are intended to represent broad mechanisms, or ‘levers’, that could feasibly be influenced or modified to improve conservation of marine biodiversity and ecosystems over the course of the next 10 years (2021–2030) (see Nash et al. 2021 for full details of methods). We then mapped umbrella drivers on two axes: 1) degree of *impact* on marine ecosystems and biodiversity and 2) degree of *influence* that society has over the driver, as we were particularly interested in umbrella drivers central to how marine biodiversity could play out in the future (high impact) and that society had the potential to influence (high influence).

Using the umbrella drivers with both high impact and high influence, we then forecast a likely ‘business-as-usual’ 2030 future based on current trends (following Merrie et al. 2018), and a ‘sustainable 2030’ future, in line with pushing towards achieving the SDGs, that is achievable if conscious actions are taken to guide the drivers towards that more aspirational future. To do this, the group brainstormed and discussed a vision for the state of the drivers in 2030 based on our shared understanding of current trends and opportunities. Sub-groups of the author team then researched individual driver trends to inform the analysis and the description of the business-as-usual and sustainable futures for each driver. All authors then reviewed the narratives and assessed the feasibility of the futures described for 2030. We then hindcast the actions required to shift from the ‘business-as-usual’ trajectory towards the more ‘sustainable 2030’ future and continued using a ‘PESTLE framework’ to ensure the generation of actions from across a wide range of categories. Importantly, the premise was that the knowledge and technology to support the actions must already exist – i.e. that there is already the capability to affect the changes we recommend. The resulting actions were temporalized to collectively form an action pathway to achieve the sustainable 2030 future, whilst iterative revisions were made between the pathway and the narrative of the sustainable future, to ensure they were realistic and technically achievable, in the judgement of the author team. It is thus important to note that the development of the scenarios, actions and pathways was not linear, but rather was iterative to ensure internal consistency (Fig. 1). Please also refer to Supplementary Table 1 for further clarification of the methodology and the scope of the paper.

Three important considerations affected what was considered within the scope of our methodological approach. 1) We note that up to and beyond 2030, the driver with the greatest impact on global marine ecosystems and biodiversity is anthropogenic climate change (Cafaro 2021; IPCC 2019; Trisos et al. 2020). Consequently, cutting greenhouse gas emissions is the action with the greatest potential benefit to the state of global marine ecosystems in the long term. Given the ‘known’ pathway to address impacts associated with climate change (e.g. IPCC 2019), and the necessity to focus on outcomes that are attainable and actionable within the next decade, we primarily examine how to reduce other impacts on marine life (e.g. resource exploitation) and increase the resilience of marine ecosystems to adapt in the face of ongoing climate change. However, our suggested actions in no way lessen the critical importance of reducing emissions without delay nor the transformations needed to supress warming in line with the Paris Agreement (Schleussner et al. 2016). 2) Many of the challenges addressed by the other papers in this special issue also affect marine ecosystems and efforts to conserve them. Where there was overlap between the challenges, this affected the level of detail we considered on those aspects of our challenge on safeguarding marine life, and we refer to those papers for additional insights and solutions. For a detailed articulation of potential actions to support mitigation of, and adaptation to, climate change in marine systems, please see Trebilco et al. (2021, this issue) and Melbourne-Thomas et al. (2021, this issue). Likewise, anticipated global trends in the demand for seafood and other products, such as energy and minerals, and the growth of activities to meet such demand will significantly impact the conservation of marine biodiversity and ecosystems into the future. These topics are discussed in full in Farmery et al. (2021), Bax et al. (2021) and Novaglio et al. (2021) in this issue. Increased pollution due to human activities is another key factor influencing our ability to conserve biodiversity and is extensively considered in Willis et al. (2021, this issue). Societal and institutional mechanisms that influence the fate of marine biodiversity, which we consider here only briefly, are explored in more detail elsewhere in this issue, and include ocean literacy Kelly et al. (2021) and ocean governance Haas et al. (2021), in addition to Indigenous rights, access and management Fischer et al. (2021).

Lastly and most importantly, 3) we note that the scenarios we describe are just two of many possible futures, and that the experiences and worldviews of the co-authors influence decisions on which drivers and actions to focus on. As such, our vision for the future presented here is likely to differ from those developed by other author groups, and our results should be interpreted within that context. We have nevertheless tried to make our vision relevant to a global audience. The goal here was not to give a prescriptive vision for the future, but to inspire thought, discussion and action, to which others can add their own visions for a better future for marine ecosystems and biodiversity.

## Results

### Drivers of marine ecosystem conservation outcomes and alternate futures for the year 2030

**Fig. 2:**
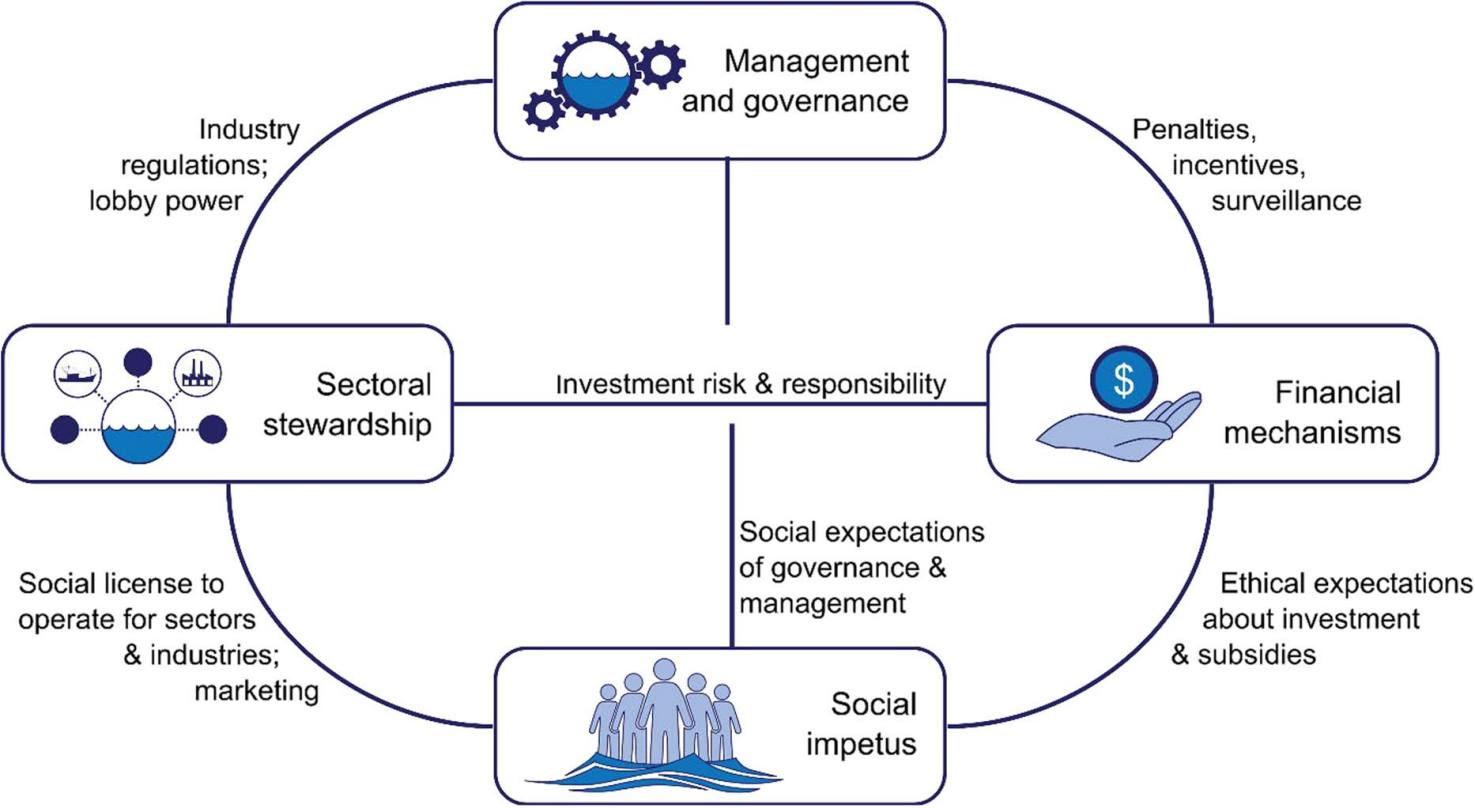
Schematic highlighting the relationship between the four key drivers of change with high potential for both impact and influence, on the fate of conservation of marine biodiversity and ecosystems by 2030

We identified four key umbrella drivers of marine conservation: (i) financial mechanisms, (ii) sectoral stewardship; (iii) management and governance; and, underpinning these first three drivers in many ways, (iv) social impetus for safeguarding marine ecosystems (Fig. 2). These drivers can negatively or positively affect conservation outcomes and thus represent potential axes of impact. Importantly, these drivers interact with each other and have feedbacks between them. Change in all four drivers is required to reach a more sustainable future. For the business-as-usual future, the drivers are assumed to progress throughout the next decade along their current trajectories, and may include both potentially positive or negative changes. Whereas for the sustainable 2030 future, the drivers evolve along aspirational but achievable trajectories. Below we describe the current state and trends of the four drivers and indicate how they may be influenced throughout the upcoming decade to shape the two alternate futures for the year 2030.

#### Financial mechanisms

Financial or economic mechanisms are powerful drivers of conservation, and routinely influence the management and conservation of marine ecosystems around the world (Innes et al. 2015; Rydén et al. 2020; Sumaila et al. 2021). Typically, however, global economic systems are characterised by processes that prioritise profit and exploitation of resources over the long-term conservation of biodiversity and associated ecosystem services (e.g. Sethi et al. 2010). Greater emphasis on marine ecosystem health (and the benefits and services provided by those ecosystems) is needed when balancing economic returns with environmental cost.

Broadly speaking, development and application of financial mechanisms are influenced by each of our drivers, including social and sectoral demand for “green” solutions; governance incentives, disincentives and requirements for accountability and best practice; as well as changes from within the finance sector. We note that shifting to a circular economy (Stahel 2016) will help reduce impacts on marine life but will not be achieved within a decade. Below we highlight specific financial resources and mechanisms that can be changed to improve marine conservation.

Financial resources and tools can be used to drive positive change for marine environments and redistribute pressure on marine resources, reduce stressors, and support ecosystem restoration; however there is currently a large marine conservation funding shortfall (e.g. it has recently been estimated that an extra US$149.02 billion per year is required to achieve SDG 14, Johansen and Vestvik 2020). At present, the dominant mechanism for financing conservation activities is via grants from governments or philanthropic sources (Bos et al. 2015). These grants can be sporadic in nature and allocated on timescales too short to fully achieve optimal conservation outcomes, or for the societal benefits of the conservation activities to be felt (Bos et al. 2015). To better conserve marine environments, greater security of funding sources and mechanisms is required (Bos et al. 2015; Fujita et al. 2013; Johansen and Vestvik 2020; Tirumala and Tiwari 2020).

Market-based mechanisms for raising such revenue can involve incentives and disincentives; for example investment in ecosystem services such as blue carbon and fees, taxes or fines for the use (or misuse) of marine services, resources, or spaces. Other financial disincentives include biodiversity offsets or performance bonds paid as a security against harming ecosystems (Bos et al. 2015; Deutz et al. 2020). Overall however, most mechanisms are under-utilized or poorly applied. For example, some subsidies for commercial fishing support activities that are otherwise unprofitable, and waste capital (estimated at US$35 billion in 2009, Sumaila et al. 2016), and which could be better employed to boost sustainability and efficiencies in the sector (Schuhbauer et al. 2017, 2020). Many ecosystem services remain unvalued or undervalued (e.g. nutrient cycling, biodiversity supporting fisheries productivity), and rarely do users pay for all the services they financially benefit from (Fujita et al. 2013; also see Haas et al. 2021).

Safeguarding marine environments therefore requires an urgent recalibration from within the financial sector, and an alignment with climate change mitigation commitments and sustainability goals (e.g. Schelske et al. 2020). Restructuring investment markets and reducing risks associated with private-sector investment in marine sustainability are critical for this (e.g. Fujita et al. 2013; Tirumala and Tiwari 2020). One mechanism developed recently is ‘blue bonds’, which enable developing countries to attract and leverage philanthropic investment to refinance national debt and fund marine conservation and sustainability projects (The World Bank Group 2020; TNC 2020). New financial mechanisms and frameworks will be required to scale up investment and ensure stable funding for marine conservation and sustainability, but must also be implemented transparently and with appropriate representation (Alexander et al. 2021; Tirumala and Tiwari 2020). This might include greater involvement of the private sector and a suite of financial mechanisms including, for example, biodiversity offsets, paying for use of ecosystem services, and blended finance (Deutz et al. 2020; Johansen and Vestvik 2020).

#### Sectoral stewardship

Terrestrial and marine industries are affecting and driving change in marine ecosystems. Many terrestrial agricultural, silvicultural, and manufacturing industries contribute to the input of harmful sediments, chemicals, and nutrients into marine environments, while tourism, construction and extractive industries (such as fishing, oil and gas and mining) also directly and indirectly impact species, habitats, and ecosystems (Luypaert et al. 2020). The scope of this driver is focused on the role that industries (including individual companies and industrial organisations) play in shaping and contributing to interactions with marine ecosystems and conservation outcomes. Sectoral decisions affecting interactions with marine ecosystems can broadly be influenced by management and governance structures, social demand for sustainable products and services, and financial market conditions, as well as by leadership from influential industry bodies and actors.

The nature and strength of sectoral stewardship is influenced by the regulatory environment for industries whose actions affect marine ecosystems. Regulation and mitigation efforts to reduce the impacts of industry interactions in the marine environment are typically reactive, with the result that interventions are often implemented too late to be effective, or need to be in place for extended periods in order to be effective (e.g. Constable et al. 2000). Decision making is often siloed within industries, such that cumulative effects – from other industries and drivers – are often inadequately considered in regulation (Link and Browman 2017; Stephenson et al. 2019). This is especially critical in coastal zones, where the vast majority of marine activities occur, and where terrestrial and marine activities often interact to produce significant environmental impacts (Bax et al. 2021; Willis et al. 2021, both this issue). However, siloed decision-making is also of increasing concern in offshore waters, where the blue economy is expanding (Novaglio et al. 2021). Implementation of measures that might assist in the recovery of ecosystems can be slow and ineffective because of competing interests in these regions, and although most activities are monitored to some extent, many lack adequately designed or enforceable regulation frameworks (Cinquemani 2019; Hofman 2019). Implementation of integrated, ecosystem-based management requiring monitoring of impacts and transparent, balanced consideration of trade-offs can therefore empower sectors to make sustainable changes (Stephenson et al. 2021).

International, multinational, and transnational ownership structures can enable corporations to avoid governmental oversight and regulations, often at the cost of environmental integrity (Folke et al. 2019; Sterner et al. 2019). This influence can undermine the setting of effective conservation measures, particularly where those measures might have economic impacts for industries. Conversely, this also means that large transnational corporations and industries can have disproportionate power to stem declines in marine biodiversity and promote shifts towards more sustainable outcomes (Folke et al. 2019; Virdin et al. 2021). Many businesses and industries are increasingly becoming more active in addressing environmental concerns and conservation, often as a response to consumer demand (GSIA 2018). However, difficulty assessing claims to sustainability and concerns over “green-washing” act as a barrier to greater investment in green businesses, and curbs the growth and potential for greater positive contributions from industries to conservation outcomes (de Silva et al. 2019; Lewis et al. 2016; Walker and Wan 2012). Increasing transparency and accountability, e.g. with development of standard metrics for assessing environmental impacts, could therefore greatly influence the market landscape and decision-making within industries.

#### Management and governance

Approaches to ocean management and associated governance and legal frameworks have evolved incrementally as disparate responses to specific environmental issues (e.g. pollution from land-based sources), into increasingly integrated and strategic approaches, such as integrated coastal zone management (ICZM) (e.g. Glaeser 2019). Modern approaches to managing marine biodiversity now incorporate many different tools, operating at a range of scales. Conservation management frameworks can comprise top-down approaches in which policy and legislative instruments implement international conventions and agreements and meet national priorities; or bottom-up approaches including customary or Indigenous, ecosystem-based and stakeholder-based approaches to resource management. Many frameworks seek to integrate a mixture of top-down and bottom-up approaches, with varying levels of social and ecological ‘success’ (e.g. Singleton 2009).

Several legally-binding international conventions and agreements focus on reducing anthropogenic impacts on the marine environment (see Table 1). They vary in many ways including in their compliance mechanisms, state party membership and the political dynamics that accompany their implementation. This regime is extremely complex, comprising autonomous, non-hierarchical and partially-overlapping institutions, agreements, and authorities (Alter and Raustiala 2018); and despite the number of legal instruments and institutions, marine biodiversity and ecosystem health have continued to decline (UN 2020). The international regime for marine environmental governance is facing a host of new challenges, including physical changes such as ocean acidification and warming, and challenges to the fitness and capacity of the governance regime itself. For example, resource distributions and global priorities are increasingly contested, and global and regional geo-political dynamics are changing, exacerbating the complexity of marine environmental governance (Spalding and de Ycaza 2020). It is also becoming more difficult for current international governance regimes to achieve an effective balance between implementing strong, clear and enforceable obligations on the one hand, and enhancing the kind of broad, global participation that will be required to address global marine environmental problems. Aspirational targets such as the Aichi Targets under the Convention on Biological Diversity, and the United Nations SDGs, may play an important role in guiding future priority setting and building momentum for global marine conservation (e.g. Spalding and de Ycaza 2020). However, robust, inter-governance regime coordination mechanisms and strong, effective action at national and regional levels will be crucial to improving the success of marine conservation and governance in the future (e.g. Grip 2017).

**Table 1:** An overview of the international legal and policy framework for conserving marine biodiversity

Legal/policy instrument	Important objectives and targets for biodiversity conservation	Legal tools and principles for implementation	Compliance mechanisms
UN Convention on the Law of the Sea (UNCLOS)^1^	Sustainable use and governance of activities on the oceans	Establishes normative principles such as: environmental impact assessment; sustainable development; and ‘polluter pays’	**STRONG** **State parties must develop national laws for implementation**
	Conservation, preservation and protection of the marine environment from human activities	Obliges States to take measures to protect and preserve the marine environment	**Disputes can be negotiated, subject to arbitration, or (unilaterally) litigated in International Tribunals**^**2**^
UN Fish Stocks Agreement (under UNCLOS)^3^	Obliges states to cooperate in conserving marine fishery resources	Establishes normative principles such as: sustainable use of fisheries resources; prevention of harm from pollution; ecosystems and biodiversity conservation; and the precautionary principle	**STRONG** **State parties responsible for compliance through cooperation. No sanctions mechanism**
	Focuses on effective management of highly migratory or straddling fish stocks across EEZs and/or an EEZ and the high seas	Supports legal/policy mechanisms for formal regional cooperation between States	**Adopts UNCLOS dispute resolution mechanisms including arrangements for compulsory procedures and binding decisions**
	Promotes optimal utilization of fisheries resources within and beyond EEZs		
CAMLR Convention (under the Antarctic Treaty System)^4^	To conserve marine life and environmental integrity in and near Antarctica	Binds parties to the *Protocol on Environmental Protection to the Antarctic Treaty* and its annexes	**STRONG** **Creates a system of ‘observation and inspection’ of activities**
	Facilitating research and studies on Antarctic marine living resources and ecosystems	Creates an Ecosystem Monitoring Program for effects of fishing and harvesting in Antarctic waters	**Disputes may be referred by consent to negotiation, arbitration or resolution by the International Court of Justice (ICJ)**
		Creates MPAs	
UN Convention on Biological Diversity (CBD)^5^	Conservation of biological diversity and the sustainable and equitable use of its components	Establishes normative principles such as: species, habitat and ecosystem-based conservation for marine biodiversity	***MODERATE*** *State parties should develop national laws for implementation*
		Creates legal tools such as marine protected areas	*Parties responsible for biodiversity within their own borders*
			*Disputes can be negotiated, referred to arbitration or, by agreement, referred to the ICJ for resolution*
International Convention for the Prevention of Pollution from Ships (MARPOL) ^6^	Minimising the environmental impact of maritime activities	Imposes strict controls on the discharge of different categories of substances that originate from ships, from sewage and garbage to noxious substances and oil	***MODERATE*** *Creates penalties and sanctions for violations, implemented through national laws in the jurisdiction in which the violation occurred*
	Preventing and minimizing accidental pollution from ships and damage arising from routine operations		*State parties are encouraged to develop national laws to improve compliance*
Convention on Migratory Species (CMS) ^7^	Encouraging range states to cooperate on the conservation of migratory wild animals and their habitats	Articulates key principles including research, immediate protections and multiple state agreements for migratory species protection	***MODERATE*** *State parties are encouraged to develop national laws to improve compliance*
		Creates Appendices that list endangered migratory species and migatory species conserved by international agreements to prioritise conservation measures by state parties	*Disputes can be negotiated or referred, by consent, to arbitration*

Each of the conventions and associated agreements listed are relevant to the 2030 future scenarios described in the section ‘Plausible Futures for 2030’. Bold indicates strong compliance mechanisms and Italics moderate compliance mechanisms

^1^United Nations Convention on the Law of the Sea, adopted and opened for signature 10 December 1982, entered into force 16 November 1994, 1833 UNTS 3 (UNCLOS)

^2^Eg a country can take a dispute with another country to the International Tribunal for the Law of the Sea, International Court of Justice (ICJ) or special arbitration tribunals constituted in accordance with the Convention

^3^The Agreement for the Implementation of the Provisions of the United Nations Convention on the Law of the Sea of 10 December 1982 relating to the Conservation and Management of Straddling Fish Stocks and Highly Migratory Fish Stocks. adopted 4 August 1995, entered into force 11 December 2001, 2167 UNTS 3 (‘UN Fish Stocks Agreement’)

^4^The Convention for the Conservation of Antarctic Marine Living Resources, opened for signature 1 August 1980, entered into force on 7 April 1982

^5^Convention on Biological Diversity, adopted 5 June 1992, entered into force 29 December 1993, 1760 UNTS 79

^6^International Convention for the Prevention of Pollution from Ships, adopted 2 November 1973, entered into force 2 October 1983; and its Protocol of 1978, adopted 17 February 1978, entered into force 1 October 1983, 1340 UNTS 62 (MARPOL)

^7^Convention on the Conservation of Migratory Species of Wild Animals, opened for signature 23 June 1979, entered into force 1 November 1983, 1651 UNTS 333

Beyond consideration of fishing effects on some biodiversity components in high seas areas (e.g. conservation measures implemented through Regional Fisheries Management Organisations), there remain significant gaps in legal and management arrangements for biodiversity conservation in these regions. Negotiations are currently underway with a focus on developing an international legally binding treaty on marine Biodiversity in areas Beyond National Jurisdiction (the BBNJ Treaty) (Ban et al. 2014; Humphries and Harden-Davies 2020). Once finalised, this will go some way to filling such governance gaps. Biodiversity conservation frameworks and action plans have also been established at regional scales, including under the UNEP Regional Seas Programme, obliging state parties to either collectively or individually set up or enhance measures to protect fragile ecosystems (e.g. in the Southern Ocean and Western Indian Ocean regions, see Oral 2015).

Most developed and developing countries have national and regional governance frameworks for marine conservation and sustainability; however, their implementation varies widely. This variation can be attributed to several factors including differences in policy priorities, diverse approaches to ocean management, and capacity challenges that hinder effective governance (see Islam and Shamsuddoha 2018). Limitations in capacity and capability have resulted in uneven outcomes for marine species and ecosystems, and can undermine conservation or management efforts where species and ecosystems are shared across jurisdictions. It can also limit the ability of countries to effectively take part in negotiations, resulting in geographic disparity in overall achievement of priorities for conservation of the marine environment (Halvorssen 2019). Marine conservation may also be given a relatively low priority when compared to other development priorities. For example, recent research demonstrates that a majority of countries prioritise socio-economic SDGs over the marine environment-based SDG 14 and that efforts to achieve SDG 14 are allocated less funding than any other SGD priority (Custer et al. 2018; Johansen and Vestvik 2020).

Although many frameworks across numerous countries aspire to incorporate integrated approaches to ocean management (such as marine spatial planning, ICZM and ecosystem approaches), in most cases management frameworks still only address single sector activities (e.g. fishing, energy extraction, shipping). While this simplifies priority setting and actions to achieve those priorities, a lack of integration can result in conflicting priorities between sectors and uneven access to ocean resources, including cultural heritage (Jones et al. 2016). This can lead to patchy outcomes for the conservation of species, communities and ecosystems, particularly where they are affected by cumulative impacts from multiple sectors and across multiple jurisdictions. Opportunities for more sustainable governance exist (Haas et al. 2021; Rudolph et al. 2020) and ultimately, this driver can be influenced by social pressure, including the expectation that marine spaces and biodiversity will be sustainably managed, sectoral support for ecosystem-based management, and through securing sufficient funding to implement and sustain integrated management.

#### Social impetus for marine ecosystem conservation

Social impetus for conservation has the potential to generate tremendous power for change. However, industrialisation and globalisation have resulted in a general loss of connection between people and environments and ecosystems (see also Kelly et al. 2021, this issue). Communities across the world depend directly and indirectly on marine ecosystems (see also Nash et al. 2021b, this issue); however, for many people conservation of marine biodiversity is a luxury, for example when the only options for accessing protein or generating a livelihood are based on unsustainable activities (Adams et al 2004; Cinner et al 2014; Glaser et al 2018). Addressing inequality, poverty and social justice is therefore critical for influencing social impetus for marine conservation (see also Alexander et al 2021, this issue).

In many cases, individuals are unaware of the impact their everyday actions have on the health and function of marine environments and the ecosystem services they provide (Bleys et al. 2017). However, greater interpersonal connectivity and access to knowledge seems to be increasing awareness of some impacts and issues facing the marine environment (Boulianne et al. 2020). Importantly, social connection – the shared emotional relationships between individuals or cohorts (Clark et al. 2017; Seppala et al. 2013) – centred on environmental sustainability is needed for awareness of marine environmental issues to translate to social impetus for sustained conservation action on conservation issues. Social connection can also help promote a shared identity and set of norms and values around concepts such as ‘ecological sustainability’ (e.g. such as those related to jobs and money). Further, a lack of connection and trust can hamper the social understanding and accurate communication of these often-complex issues (Ives et al. 2017).

Currently, many of the environmental issues that attract considerable public and media attention and action (such as oil spills and reduction in single-use plastics, Eddy 2019; Edgar et al. 2003) tend to be singular, easily observed problems for which solutions can be simply articulated (also see Kelly et al. 2021, this issue), rather than the far more damaging, complex and cumulative impacts that marine ecosystems face. Advancing ocean literacy and empowering people to make informed choices that support marine conservation (e.g. through access to information) are particularly important for influencing social impetus (Kelly et al. 2021; Nash et al. 2021b, this issue). Where conservation efforts result in reduced delivery of benefits, substantial structural resistance to those efforts can occur (Alexander et al. 2021 this issue). Social impetus for conservation is more likely to be strong where conservation outcomes can be linked to proximal economic benefits and societal survival (Kauder et al. 2018). However, linking conservation goals and strategies with social dependencies on the services marine ecosystems provide can be a powerful mechanism for creating collective action (Barnaud et al. 2018).

### Plausible Futures for 2030

#### Business-as-usual 2030 – ‘too little, too late is tragically common’

**Fig. 3:**
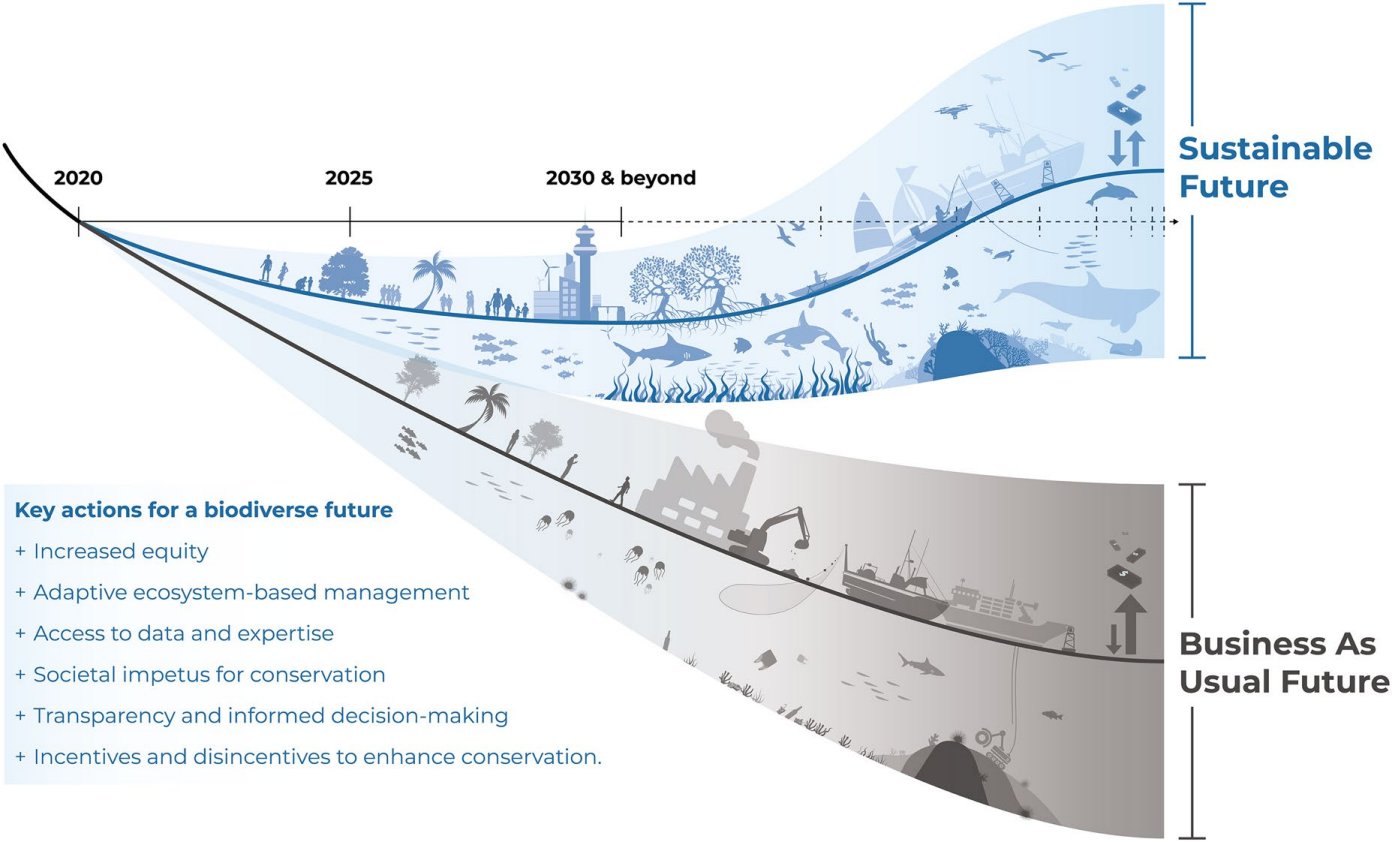
The trajectories of marine biodiversity change we envisage under a business-as-usual scenario (grey line) and under our more sustainable but technically achievable scenario (blue line). The y-axis represents marine biodiversity and the x-axis represents time. Figure format inspired by a graphic by A Islaam, IIASA

Along the business-as-usual trajectory towards 2030, there will certainly be progress made relative to the beginning of the decade, with increased implementation of conservation measures (e.g. improved design and establishment of MPAs, improved monitoring through use of technology), improved management and regulatory frameworks with associated reductions in some pressures and steady increases in habitat restoration (see below). However, much of the progress in conservation outcomes is geographically biased and overall the trajectory for marine ecosystem health continues on a decline (grey line, Fig. 3). Positive progress, and the actions that facilitated them, seem likely to be too sporadic and reactive to ensure the widespread improvements needed in many regions; this is driven largely by unequal availability (and thus inequality) of financial resources and expertise devoted to improving conservation outcomes. Decision-making and drivers of conservation outcomes and marine ecosystem health are still mostly siloed and isolated from one another, leading to insufficient collaboration and consideration of cumulative impacts. Ultimately, it seems that progress and concordant conservation benefits will be best summarised as ‘too little, too late,’ and continue to be obstructed by commercialisation of exploitation. Under this scenario, by 2030:

- Implementation of integrated, marine spatial planning has increased, but is undertaken in approximately only 30% of EEZ’s globally (IOC-UNESCO 2017, 2018)
- Social impetus for safeguarding and recovering marine ecosystems has increased sporadically (e.g. Agardy 2005; Hawkins et al. 2016; Kelly et al. 2018; Wynveen et al. 2014)
- Management of the marine estate remains predominantly siloed, reactive, and often lacks strategic conservation goals (e.g. Alvarez-Romero et al. 2018)
- Lobbying continues to impede the development and/or implementation of new financial or regulatory mechanisms to mitigate impacts on marine ecosystems (e.g. Etzion 2020; Folke et al. 2019)
- Increased demand for sustainable products and services drives sporadic improvements in some industries/companies, but this has yet to trigger a broader shift in practices that improve or minimise harm to marine environments (e.g. Lim 2017)
- Geographic bias in marine ecosystem research, management, and conservation continues (e.g. Alvarez-Romero et al. 2018; Di Marco et al. 2017)
- Negotiations for a new UN treaty on Biodiversity Beyond National Jurisdictions (BBNJ) have proceeded very slowly (noting the effect of the coronavirus pandemic on the scheduling of conferences of the parties and intersessional activities) and seem increasingly unlikely to result in strong, legally binding conservation obligations (Tiller et al. 2019), even as extractive industries continue expanding in areas beyond national jurisdiction.

#### Sustainable 2030—‘building momentum for conservation success’

In the sustainable 2030 scenario, while there still remains considerable room for improvement, the overall trajectory of ecosystem decline present at the beginning of the decade has been arrested (blue line, Fig. 3), with increasing momentum and a rapidly growing number of success stories resulting in clear reversal in some regions and ecosystems (Abelson et al. 2016). Pressures on many marine environments have declined due to more collaborative and proactive regulation, aided by increased action to address the inequality of resources available to support regulation and management. Indeed, well-resourced, cross-disciplinary integrated management emerges as a cornerstone of the positive conservation outcomes that are occurring, and which have taken place at all scales, from local to international. Under this scenario, by 2030:

- Integrated, ecosystem-based management of marine ecosystems has been widely implemented (e.g. Delacámara et al. 2020; Link and Browman 2017; Stephenson et al. 2021; Stephenson et al. 2019)
- There is increased social impetus and empowerment for the safeguarding of marine ecosystems (e.g. Hawkins et al. 2016; Kelly et al. 2018)
- Community-members and decision-makers are better informed about the importance of marine ecosystems and positive practical actions they can take (e.g. Artelle et al. 2018; Kaplan-Hallam and Bennett 2017)
- Growing interdisciplinary collaborations and cross-sectorial regulations reduce negative impacts on marine ecosystems and promote a shift towards a more circular economy (e.g. Stahel 2016; Kirchherr et al. 2017)
- Greater emphasis on environmental impacts in triple-bottom-line accounting, in conjunction with financial mechanisms, to support and rebuild marine ecosystems (e.g. Bos et al. 2015; Dichmont et al. 2020)
- Capacity-building in under-resourced communities decreases regional inequalities in development and implementation of integrated spatial management (Alvarez-Romero et al. 2018; IOC-UNESCO 2017)
- Improved ecological monitoring and forecasting, and the transfer of such information, both of which enable more proactive, flexible, and adaptive management (e.g. Pendleton et al. 2020)
- Improved monitoring, evaluation and adaptation of management strategies and plans (Ehler 2014; IOC-UNESCO 2017)
- Negotiations for a new UN BBNJ treaty have proceeded slowly (noting the effect of the coronavirus pandemic on the scheduling of conferences of the parties and intersessional activities) but seem increasingly likely to result in legally binding conservation obligations, and important States have indicated that they intend to ratify the treaty.

### Pathway to achieving a sustainable future

We identified a series of actions, each associated with one or more of our drivers, that together could form a pathway for achieving a more sustainable 2030 future for marine biodiversity and ecosystems (Tables 2, 3, 4, 5). These actions are grouped in four categories, which correspond with overarching goals for our pathway (listed below). Within each category we identify when actions commence on the spectrum from short-term (2021–2025), medium term (2025–2030) and long-term (2030 and beyond). We also identify who, amongst governments, industry and research institutions, might need to undertake those actions, as well as describing the scales (local, regional, global) that are applicable for each action. For each action we also specify the driver (or in some cases two drivers) which that action addresses.

**Table 2:**
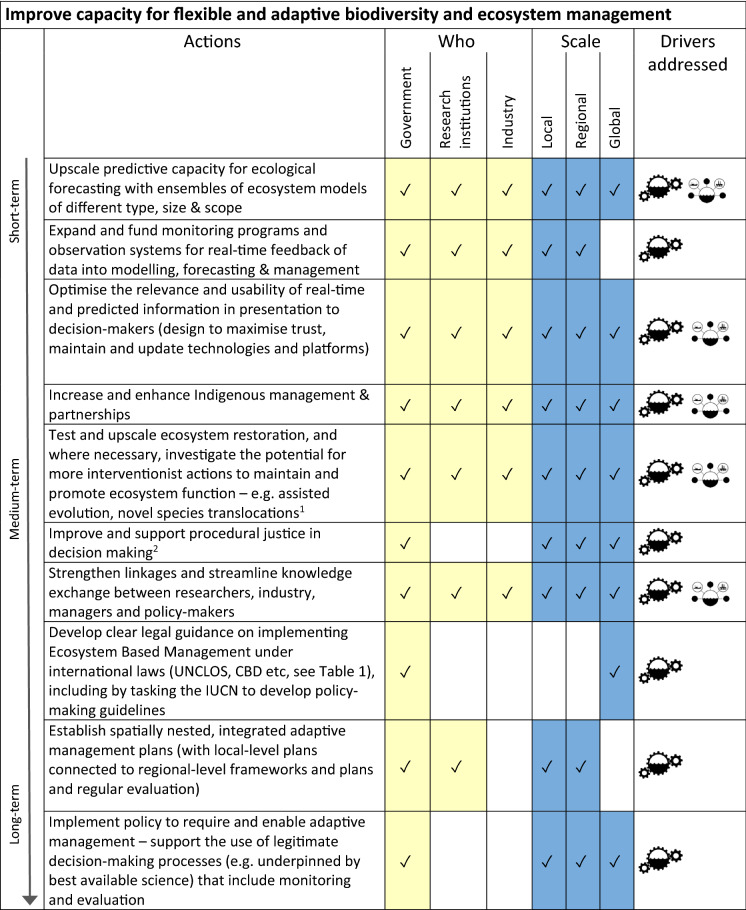
Actions for improving capacity for flexible and adaptive biodiversity and ecosystem management.  = Management & Governance;  = Sectoral Stewardship

^1^For example see Gattuso et al. (2018), IPCC (2019), Duarte et al. (2020)

^2^See Alexander et al. (2021, this issue)

**Table 3:**
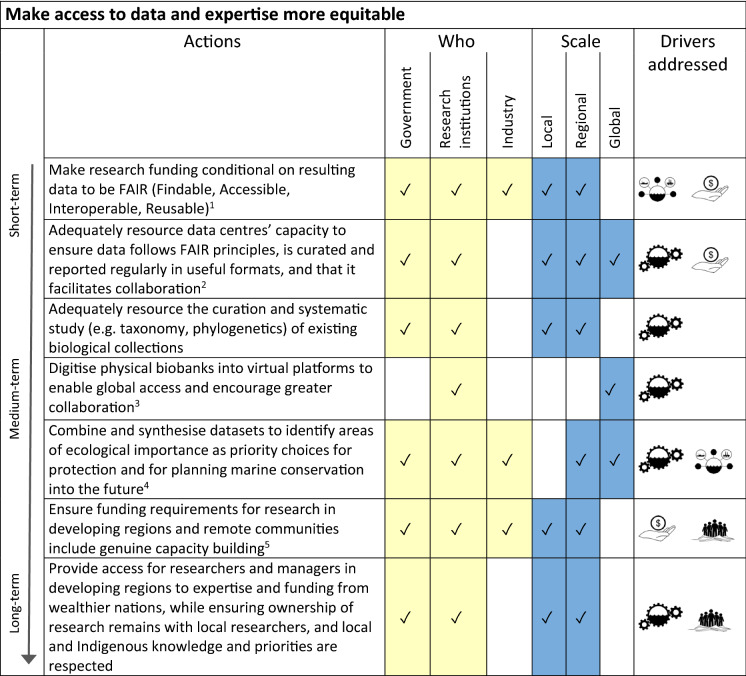
Actions for making access to data and expertise more equitable.  = Management & Governance;  = Sectoral Stewardship;  = Social Impetus;  = Finance

^1^See Wilkinson et al. (2016)

^2^For example see Edgar et al. (2016), https://schema.org/, https://datasetsearch.research.google.com/

^3^For example Otlet (Green et al. 2019), Atlas of Living Australia (http://www.ala.org.au)

^4^For example see Hindell et al. (2020)

^5^For example the Australian Centre for International Agricultural Research (ACIAR) supports and funds thousands of agricultural and aquaculture projects by building capacity of individuals and institutions in-country (https://aciar.gov.au/cross-cutting-areas/capacity-building)

**Table 4:**
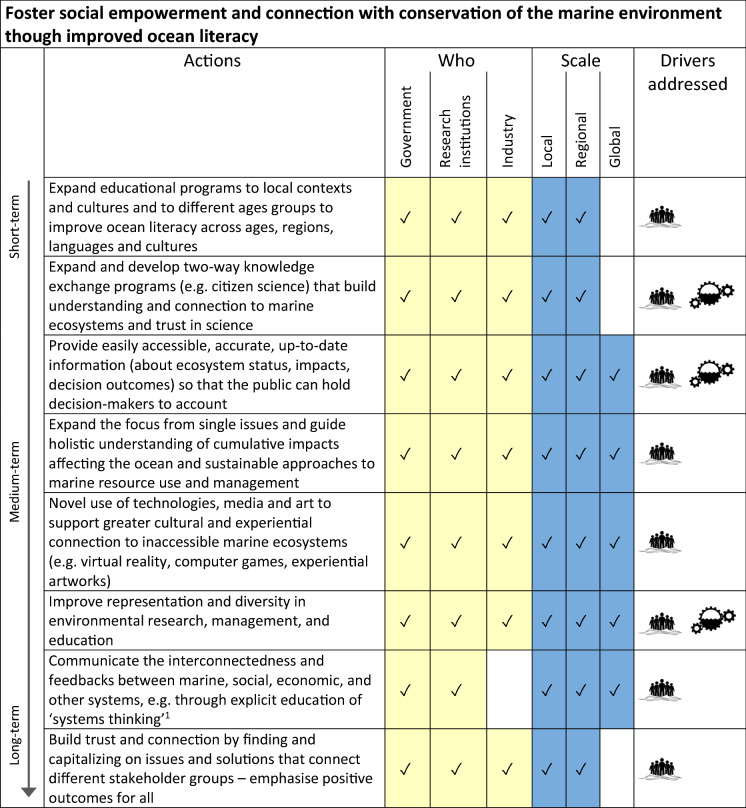
Actions for increasing societal impetus for conservation of marine biodiversity through improved ocean literacy and communication.  = Management & Governance;  = Social Impetus;

^1^see also Kelly et al. 2021, this issue

**Table 5:**
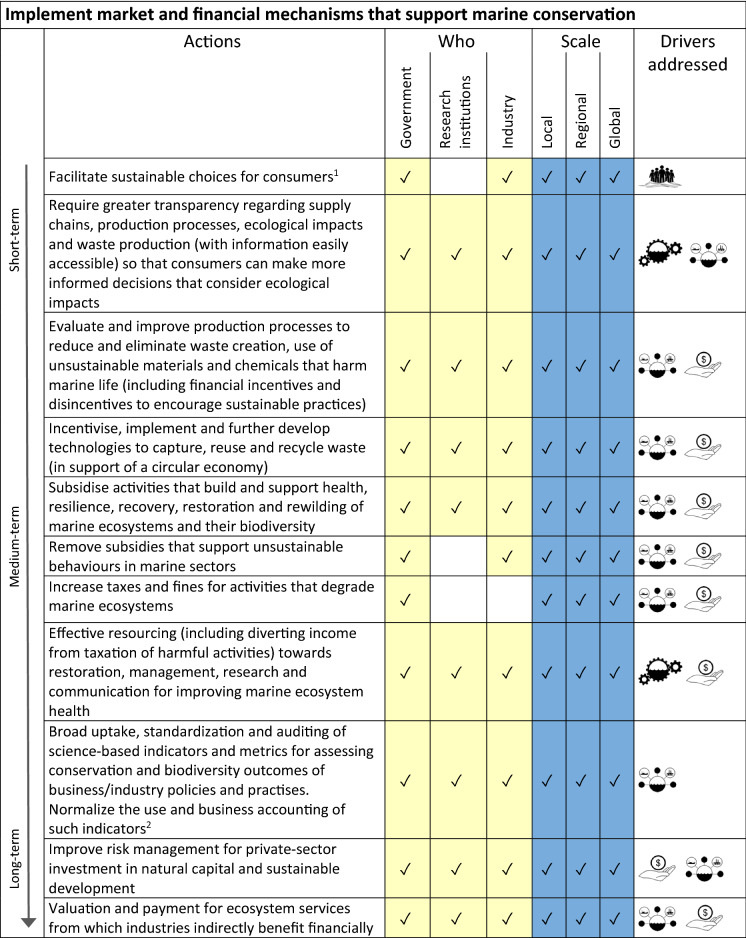
Actions for identifying and implementing market and financial mechanisms to reduce impacts and support conservation.  = Management & Governance;  = Sectoral Stewardship;  = Social Impetus;  = Finance

^1^For example, the Oceanwise Program (seafoodwatch.org/)and Seafood Watch (www.seafoodwatch.org/)

^2^See Vörösmarty et al. (2018),Addison et al. (2019)

The four categories/overarching goals for our sets of actions within the pathway are:

1. To improve capacity for flexible and adaptive biodiversity and ecosystem-based management in the marine environment (Table 2; see also Haas et al. 2021, this issue). The actions in this category mostly address the management & governance driver described above.
2. To make access to data and expertise more equitable (Table 3). This includes financial mechanisms (e.g. increased funding, incentives) to make data more accessible as well as capacity building in regions with fewer resources to research and implement adaptive management. Actions in this category collectively address all four of our drivers.
3. To foster social empowerment and connection with conservation of the marine environment through improved ocean literacy (Table 4; see also Kelly et al. 2021, this issue). These actions include formal and informal education, citizen science, and mechanisms for increasing accessibility of information to the public about a) status of marine ecosystems, and b) progress in safeguarding marine ecosystems. These actions together address our social impetus driver.
4. To implement market and financial mechanisms that support marine conservation (Table 5). This set of actions consider consumer choice and transparency in supply chains (see also Farmery et al. 2021, this issue), as well as financial incentives and disincentives for industry (see Novaglio et al. 2021, this issue), and addresses all four of our drivers, but most specifically the sectoral stewardship and financial mechanisms drivers.

**Fig. 4:**
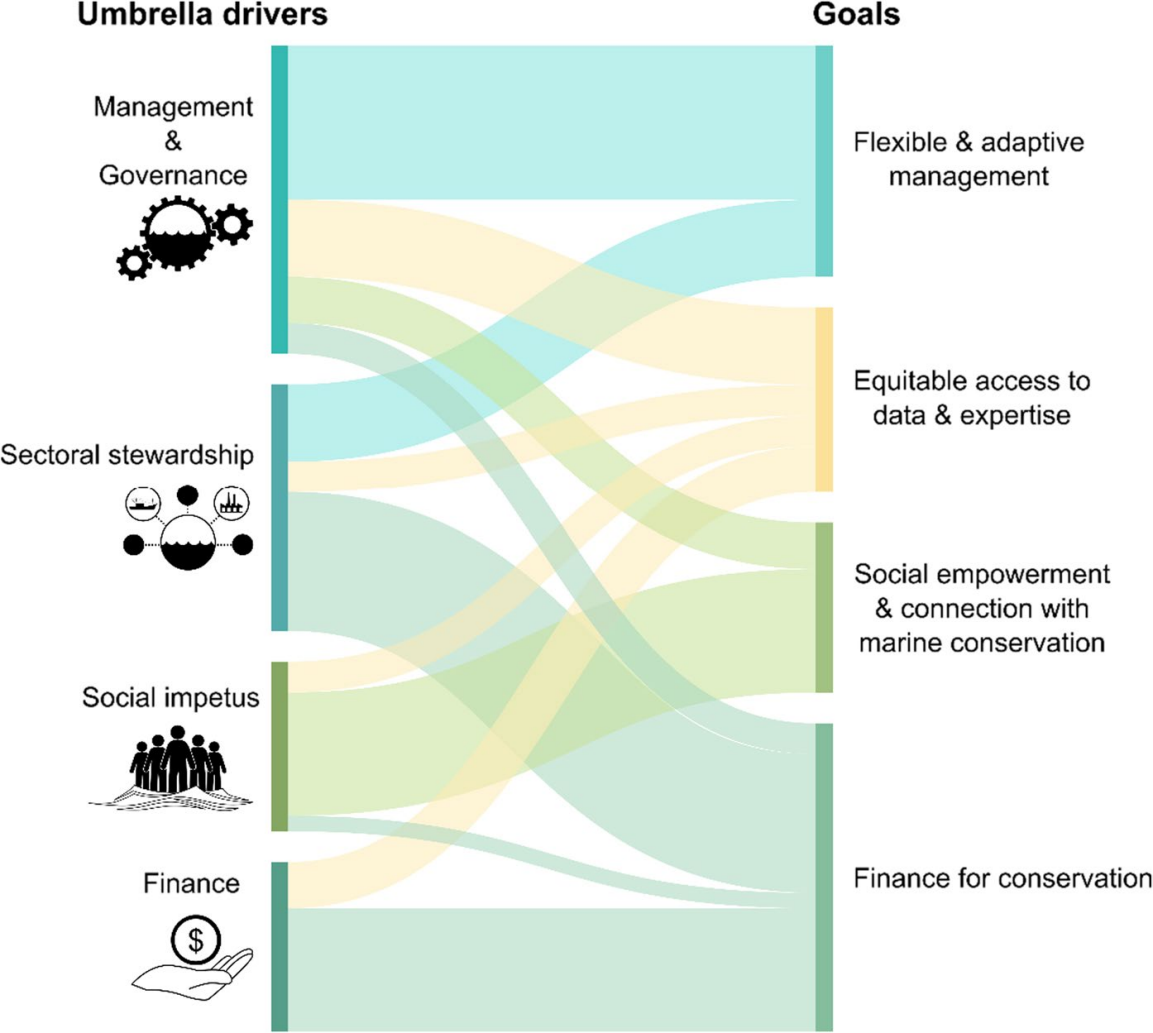
Relationships between the umbrella drivers of marine ecosystem change on the left, and our overarching goals for a more sustainable 2030 on the right. Filaments between the nodes represent the actions presented in Tables 2, 3, 4, 5, coloured according to the goal to which they primarily contribute

Relationships between the drivers and our overarching goals towards the more sustainable future are illustrated in Fig. 4. Importantly, successful examples of the implementation of many of the actions we describe already exist – which highlights that this pathway is achievable with sufficient political and socioeconomic will. We describe some examples of these ‘bright spots’ in Table 6, pertaining to a series of different habitat or biodiversity components, and summarise who undertook specific actions and at what scale, as well as the factors that enabled specific actions, to realise these examples of success.

**Table 6:** Example ‘bright spots’ for management and conservation of habitats and biodiversity, which demonstrate successful actions and illustrate ways in which the pathway for a more sustainable 2030 might be achieved. Our ‘bright spots’ represent areas and/or projects where specific actions have been taken to restore, sustain or protect marine habitats and biodiversity (e.g. as per Bennett et al. 2016; Cinner et al. 2016; Cvitanovic and Hobday 2018). These bright spots help demonstrate the potential to create a more desirable future for our oceans. In some cases, results and trends following these interventions are well established, while in other more recent actions, they are still emerging

	‘Bright spot’ example	Key management and conservation actions	Actors and the scale of actions	Key ‘enablers’ facilitating the specific conservation actions, with links to **drivers**
Land to sea linkages in the coastal zone	Queensland, Australia “Paddock to Reef” management of land-based run-off (e.g. sediments, nutrients, pollutants), to improve coastal water quality and lessen a key stressor on the region’s inshore marine ecosystems (GBRMPA 2020; State of Queensland 2018)	Cross-sectoral management and partnerships (see next column) Catchment improvement via the upgrading/modification of infrastructure, and restoration of habitats (e.g. riparian habitats, wetlands/tidal marshes, disused agricultural land) and associated ecosystem services	On-the-ground action at local to regional scales, with collaborations amongst marine and terrestrial managers and regulators, farmers and other landholders, Traditional Owners, local community groups, and scientists Regulatory reform by government at local to national scales	Management and governance – cross-sectoral and interdisciplinary Financial mechanism – market-based instruments to support investment and funding Social impetus and Sectoral stewardship – promoted by community and industry engagement
Coral reefs	Reef fish biomass, Muluk Village, Karkar Island, Papua New Guinea (Cinner et al. 2006; Cinner et al. 2016)	High local engagement in management Tenure and management and exclusion rights for the local reef Periodic closures of reef to fishing based on observational indicators of fish biomass decline	Village chiefs make the decision to close the reef to fishing based on personal catch rates and social indicators of reduced fish biomass Local community supports the decision and have lived experience that 1–2 year closures can successfully recover fish biomass and ‘catchability’	Management and governance – sociocultural governance (marine tenure, cultural taboos) Social impetus—high community dependence on coastal resources combined with lived experience of management strategy success
Temperate reefs/Kelp forests	British Columbia, Canada Kelp forest restoration via urchin removal and bolstering of sea otter populations	Regulatory protection and subsequent re-introduction of keystone predator (sea otter) Partnership with First Nations communities and governments, and commercial fishers Collaborative intervention to remove destructive and overabundant herbivore (urchin), and subsequent support of community seafood outreach programs Protection and ongoing restoration of key foundation species (i.e. the kelps) and associated biodiversity	Fisheries and Oceans Canada under the Canadian government managed the re-introduction of Sea Otters and monitor their populations Provincial, Federal, and First Nations Governments Local communities and industries Coastal management authorities	Management and governance – regulation, collaboration Financial mechanisms and sectoral stewardship – boosting commercial urchin harvesting. Return of urchin predators and kelp forests have improved fish stocks, attracted tourists as well as capturing carbon (Gregr et al. 2020; Parks Canada 2019) Social impetus – human interactions with sea otters and kelp forests by First Nations (Salomon et al. 2015)
Pelagic ecosystems	North Atlantic Swordfish fishery Fishery management via catch limits to prevent stock collapse and enable recovery	Stocks declared overfished in 1990 and recommendation for fishing effort and mortality be capped Revision of management practices (including total allowable catch, catch per unit effort, minimum landing size) based on ongoing research into population dynamics and maximum sustainable yield Public campaigns to protect swordfish Commitment to maintain stock numbers following achievement of recovery plan in 2010 (Neilson et al. 2013)	Acting Regional fisheries management organisation (RFMO): International Commission for the Conservation of Atlantic Tunas (ICCAT) NGO’s (e.g. Natural Resources Defense Council, Sea Web, The Pew Trusts and Chef’s Collaborative) lead public campaigns to promote awareness that swordfish stocks were unsustainable	Management and governance Financial mechanisms—investment in research provided the foundation for the effective conservation measures adopted by ICCAT Social Impetus—campaigns
Polar / Antarctic ecosystems	Ecosystem-based management, Southern Ocean	The Antarctic Treaty (first signed 1959) to preserve the region for peace and science, including provision to require sharing of research Convention for the Conservation of Antarctic Marine Living Resources (in 1982) adopts an ecosystem-based approach to management (Constable 2011) Precautionary principle applied to all fisheries’ catch limits, including provision for predator requirements World’s first high-seas MPA established in the southern Atlantic Ocean (in 2009). General measure for the establishment of CCAMLR MPAs adopted in 2011. World’s largest MPA established in the Ross Sea (in 2016)	Commission for the Conservation of Antarctic Marine Living Resources (CCAMLR) and the Antarctic Treaty System Scientific Committee of CCAMLR advises on best available science Members adopt conservation measures by consensus, and co-manage fisheries overlapping their EEZs International Whaling Commission (IWC), Agreement on the Conservation of Albatross and Petrels (ACAP) and other intergovernmental organisations cooperate with CCAMLR to conserve specific taxa •NGO’s (e.g. Pew Charitable Trusts, World Wildlife Fund, Southern Ocean Coalition) promote conservation actions	Management and Governance – treaties and consensus negotiation Social impetus – to protect Antarctica and its charismatic fauna from direct human impacts under climate change Sectoral stewardship – to conserve valuable fisheries and dependent species
Deep-sea benthic	Northwestern Hawaiian Ridge and Emperor Seamounts Spatial protection from detrimental bottom trawling to allow slow growing benthic fauna and habitats to recover (Baco et al. 2019)	International recognition that bottom trawling is the most detrimental to benthic habitats of all fishing methods (UNEP 2006) Use of Vulnerable Marine Ecosystem (VME) concept and indicator species for the management of deep-sea fisheries A subset of areas within the Northwestern Hawaiian Ridge and Emperor Seamount chain are protected from fishing (NOAA 2008)	Fisheries and Agriculture Organisation for the United Nations for recognising the detrimental effects of bottom trawling and setting guidelines Conservation measures by North Pacific Fisheries Commission	Management and Governance Financial mechanisms and Sectoral Stewardship—bottom trawling is unsustainable long-term
Migratory species	Humpback whale—East Australian population (International Whaling commission E1 stock), which migrates between subtropical breeding areas and polar feeding areas The population is approaching pre-exploitation numbers (Noad et al. 2019)	Slowdown and cessation of catches of this species off the East Australian coast in the 1960’s due to declining populations Domestic ban on whaling (implemented in 1979), preceeding the international moratorium on commercial whaling (implemented in 1986) Antarctic whale sanctuary established (1994) Vessel restrictions to reduce disturbance e.g. minimum distance from whales, speed, number of boats (within national waters) Ongoing work to reduce entanglements (Bolin et al. 2020) and vessel collisions (Smith et al. 2020)	International Whaling Commission (formed following recognition that whale catches were declining) Non-governmental organisations (e.g. Greenpeace, Sea Shepherd) lead public protest and anti-whaling campaigns Local and national governments created legislation protecting humpback whales in Australian waters and managing interactions between humans and whales	Management and Governance—regulation Social impetus – public campaigns Financial mechanisms – whales are more valuable alive than dead via the tourist industry (Knowles and Campbell 2011) Sectoral stewardship – to set catch limits and international moratorium

## Discussion

In this paper we have developed and outlined a technically achievable pathway to a future for marine ecosystems and biodiversity where the trajectory of ecosystem decline present at the beginning of the decade has been stemmed, and examples of conservation success, e.g. ‘bright spots’ are rapidly growing in size and number. In developing the set of actions described in Tables 2, 3, 4, 5 we endeavoured to generate a condensed list of key actions over the 2021–2030 timeframe that could form a feasible pathway towards the more sustainable future we have described for marine ecosystems globally, considering the four key drivers of change identified. Of course, in reality, there is a vast amount to be done to address the complex challenge of safeguarding marine life, and a range of factors that might influence the effectiveness and ultimate success of these actions. In the following sections we discuss five factors that we consider to be particularly important in determining capacity for action to address the drivers in a way that sets us on the pathway to a more sustainable future. These factors are: (1) connection to marine ecosystems and behavioural change; (2) empowering local communities, Indigenous management and partnerships; (3) access to accurate, up-to-date information; (4) overcoming barriers to integrated, ecosystem-based management; and (5) shifting towards a more equitable, circular economy. We acknowledge that there is a significant (and continually developing) body of literature around all five of these topics, and so in the following sections we attempt to distil the key ways in which they might influence capacity for the actions identified in our results, and hence affect the likelihood of achieving a more sustainable future for marine biodiversity. We note that addressing these factors won’t fix marine biodiversity conservation, however they can contribute to shifting our drivers within this decade, and then in the longer term (beyond 2030) these drivers will be positioned to improve marine conservation.

### Connection to marine ecosystems and behavioural change

It is not possible for all 7.8 billion people on Earth to feel deeply connected with marine ecosystems. However, actions to increase individuals’ connection with marine spaces and nature in general is likely to increase pro-environmental behaviour and attitudes, with the added benefit of improving wellbeing (Evans et al. 2018a; Kelly et al. 2021; Nash et al. 2021b; Rosa and Collado 2019; White et al. 2019). The drivers for improving human connectedness to marine environments are outlined in Kelly et al. (2021, this issue) and include education, cultural connections, technological developments and knowledge exchange and science-policy interconnections. Those authors identify five key challenges to improving ocean literacy including the need to i) expand educational programs beyond those that are youth-focused to include all components of society; ii) expand programs to local contexts and cultures to improve ocean literacy across regions, languages and cultures; iii) expand the focus on single issues and guide holistic understanding of issues affecting the ocean and sustainable approaches to marine resource use and management; iv) maximise the utility of technology in achieving ocean literacy; and v) adopt more inclusive approaches to decision making. Kelly et al. (2021) develop an ocean literacy toolkit and provide a practical pathway for improving societal connections to the marine environment, and in doing so support improved societal impetus for conservation actions.

Changing the way individuals and society consider marine ecosystems can also benefit from using diverse means of communication to reach different people in different contexts. Art, storytelling, and humour can all allow people to diverge from their normal thought processes, and to connect with information and marine environments in a different way (e.g. Curtis et al. 2012; Dahlstrom 2014; Dahlstrom and Scheufele 2018; Lenda et al. 2020; Paterson et al. 2020). Games can also be used to develop mechanistic understanding of how cumulative human actions and policies impact marine ecosystems (e.g. https://www.mspchallenge.info/), and how trade-offs in their management might affect enjoyment of marine spaces.

Leveraging behavioural science is also increasingly recognised as key to support conservation outcomes and sustainable choices and actions by consumers and communities (Bennett et al. 2017). For example, Cinner (2018) describes how, because people generally prefer to maintain the status quo, setting default options so that people need to “opt out” rather than “opt in” to sustainable options can be an effective strategy. Moreover, if people perceive environmental problems as being beyond the power of individuals to effect change, then directly facilitating sustainable choices (e.g. opt-out vs. opt-in to sustainable options), can boost the feeling of making a difference and so propel further action.

### Empowering local communities, Indigenous management and partnerships

The magnitude of the challenges facing the health and management of marine ecosystems requires innovative solutions that are capable of being implemented across all geospatial scales. Adopting a ‘bottom-up’, locally-driven approach would not only empower greater connection of local communities to their marine environments (as discussed above) but could also increase impetus for action at broader scales. However, not all communities that depend on marine ecosystems do so sustainably (e.g. Cinner et al. 2016; Dambacher et al. 2007; Glaser et al. 2018), and addressing poverty and social well-being are critical elements for achieving sustainable resource use and conservation (i.e. achieving SDG 14 depends also on achieving other SDGs) (Chaigneau et al. 2019; Coulthard et al. 2011; Nash et al. 2020). Resourcing may also be more limited at local scales and local communities are limited in the extent to which they can (independently, at least) mitigate local impacts from global challenges such as climate change. Given the variability in the capacity of local communities to safeguard marine ecosystems, and the global scale of pressures facing them, it is important to both strengthen local communities’ power to protect their local environments and also support them more effectively through integrated regional management structures. In particular, the diversity of the local communities needs to be represented in positions of responsibility in local and regional ecosystem management, monitoring and research to ensure whole-of-community support for the conservation goals and processes. If well supported, diverse decision-making teams have greater capacity to generate and explore innovative approaches to challenges and show greater thoroughness of decision-making processes and accuracy of assessments (Cheruvelil et al. 2014; Hong and Page 2004; Phillips et al. 2014), which are fundamental for improving marine ecosystem management.

The need to empower Indigenous Peoples to manage their cultural marine spaces is especially important. Indigenous Peoples have suffered from loss of territory and resources due to both the depletion of their environments by Western/global pressures and, with a few exceptions (e.g. Gwaii Haanas, and SGaan Kinghas-Bowie Seamount, both Canada), the actions of the West to conserve these now dwindling resources/environments (e.g. access to cultural fishing waters restricted due to marine reserves) (Tauli-Corpuz et al. 2020). Yet many Indigenous Peoples still have the experience and knowledge required to sustainably manage these ecosystems (see Reid et al. 2020 and the case study below). Recognition of this, along with opportunities and support (where necessary) for Indigenous Peoples to develop and formalize their own marine ecosystem management plans and objectives (Fischer et al. 2021; Mustonen et al. 2021, both this issue), is likely to result in improved marine ecosystem health at the same time as advancing equity for Indigenous Peoples (e.g. Alexander et al. 2021; Artelle et al. 2019; Ban and Frid 2018; Rist et al. 2019).

Local and Indigenous knowledge is currently under-recognised in ecosystem management activities and frameworks (Jones et al. 2020b; Ogar et al. 2020; Reid et al. 2020). Indigenous ecological knowledge is a complex system of intergenerational, experiential observations, beliefs, practices and values that has evolved as a response to interactions between culture and environment (e.g. Alexander et al. 2019; Jackson et al. 2017; Yunupingu and Muller 2009). The rich understanding Indigenous People have for their local environment is inseparable from their cultural values and practices (Frainer et al. 2020), and in many cases comprises experience and knowledge for adapting practices to large environmental change. Yet, even where Western ecosystem management frameworks try to draw on Indigenous knowledge, they often seek to separate the ecological knowledge from the cultural perspective and practices to which it belongs, and so divorce the knowledge from its context (e.g. Yunupingu and Muller 2009). Moving forward, greater emphasis on developing pluralistic knowledge frameworks and methods for bridging the separate knowledge frameworks will enable richer, and more informed management of ecosystems and people, with greater conservation and human outcomes (e.g. Alexander et al. 2019; Gavin et al. 2018; Kaiser et al. 2019; Reid et al. 2020). Importantly, the best approaches for doing so are likely to differ between cultures and environments, but a number of case studies and meta-analyses provide examples for how this can be done, e.g. Table 7, Alexander et al. (2019) (although many of these are from developed nations, i.e. Canada, New Zealand).

**Table 7:** Case study

Case study: Development of marine spatial management plans for northwest coast of Canada in partnership with First Nations’ governments *Razorclam diggers on North Beach, Haida Gwaii. Photo credit: Graham Richard* Marine spatial planning (MSP), including zoning for conservation purposes, has been a key element of marine plans developed for the Northern Shelf Bioregion (NSB), located on the northwest Pacific coast of Canada (Jones et al. 2020a). Approximately 45% of the population in the region is Indigenous, some 28 Indigenous Nations have territories in the region, and 16 are actively involved in negotiation of treaties or reconciliation agreements with Canada (see BC Treaty Commission 2020; ISC 2019). Marine Spatial Planning, in partnership with Canada, the Province of British Columbia (BC) and Indigenous Nations, has been underway since 2005 including development of i. a high level integrated marine plan, ii. four sub-regional marine spatial plans, and iii. ongoing work to design an MPA Network. A critical factor in developing these plans and initiatives has been how Indigenous groups organized themselves and established governance structures on scales conducive to planning, regardless that Indigenous rights and title occur at the scale of individual Nations (Jones et al. 2010). A similar governance structure was recently applied for marine transportation and emergency response planning as part of collaborative implementation of a federal Ocean Protection Plan (RFA 2019). MSP and marine governance efforts are seen as a facet of Indigenous reconciliation in Canada (e.g. Jones et al. 2010). i. An integrated marine plan for the NSB, the Pacific North Coast Integrated Management Plan (PNCIMA), was endorsed by federal, provincial and Indigenous governments in 2018. The plan establishes an EBM framework and identifies five priorities for implementation including governance and MPA network planning (PNCIMA 2017, 2020). Although the federal government has since identified MSP pilots in other parts of Canada, commitments to MSP in the high-level PNCIMA plan for the Pacific North Coast are minimal. ii. Beginning in 2011, four sub-regional plans and a regional action framework were developed that were endorsed in 2015 by provincial and Indigenous governments through a Marine Plan Partnership (MaPP 2020). The plans include a zoning framework based on IUCN categories that designates about 18% of the NSB as a protection management zone (PMZ). About 4% of the NSB is identified as a special management zone (SMZ) related to economic development activities. Outcomes of the Haida Gwaii Marine Plan, one of the sub-regional plans, were guided by a future scenario that outlines a marine conservation and local economy path. BC and Indigenous Nations manage activities within the PMZ to protect critical values and meet specific objectives. Progress is assessed annually based on performance measures (MaPP 2020). Plans are currently under review, with updates part of a 5-year review cycle. iii. Development of the MPA network for the NSB is progressing gradually (MPA Network 2020). An inclusive process involving key marine stakeholders was used to identify design criteria and a draft network scenario is currently being reviewed with the goal of completing the network design by 2021. The NSB planning process reflects several criteria for reconciliation identified through a review of the United Nations Declaration on the Rights of Indigenous People (Jones et al. 2020a). These include negotiation of government-to-government agreements, adequate resources for Indigenous planning and plan implementation, documentation and inclusion of traditional knowledge (Diggon et al. 2021), incorporation of Indigenous priorities into decision-making, and consent through endorsed agreements and plans. The MaPP plans achieved significant interim protection and conservation results (MaPP 2017, 2019). BC and signatory Indigenous governments are using the plans to make resource management decisions related to foreshore and marine development including forestry, aquaculture and tourism activities (e.g. Figure 4). As well, the MaPP zoning has been a key input into development of the MPA Network design. However, there are gaps related to federal jurisdiction and MSP in areas such as fisheries, oil and gas development, aquaculture and marine transportation	

### Access to accurate, up-to-date information

To be able to choose actions that support conservation of marine ecosystems, both society and decision makers need access to clear, accurate, and up-to-date information on the pressures being placed on the marine environment and solutions for reducing those pressures (see also Kelly et al. 2021, this issue). In order to provide accurate up-to-date information for decision making, information needs to be made available in real-time and in formats that are digestible to those that need and utilise this information (e.g. Lowerre-Barbieri et al. 2019). This requires improved dataflows, rapid analyses, reliable interpretation and accessible delivery. It will also require that all information generators (industry, business, society) make information accessible (Evans et al. 2018b). Ultimately, mechanisms that can bring all of these varying data sources together to provide key indicators that can be tracked and translated into forms that conservation managers can both understand and use are needed (Evans et al. 2019). Effective use of historical datasets is also needed – these data are needed to develop skill in forecasts and an understanding of what past activities have occurred in order to understand future risk. This will require digitising information that is not in digital formats, updating data in out-dated formats (that result in data not being able to be used anymore) and making these available through easy to access dataflows. Targeted efforts in this regard have been undertaken with oceanographic data (Woodruff et al. 2005). Further, large scale assessments relating to the marine environment, currently released at scales of 5 or more years, are recognising the need to provide information in more digestible formats (e.g. the interactive atlas of the most recent working group 1 assessment report of the intergovernmental panel on climate change, see https://interactive-atlas.ipcc.ch/), in ways that allow for updating of information on more frequent time scales (e.g. for example on annual time scales such as that of the World Meteorological Organisation’s state of the global climate reports, see https://public.wmo.int/en/our-mandate/climate/wmo-statement-state-of-global-climate. These efforts need to be expanded to include information on marine ecosystems.

Methods for communication can include technological tools such as environmental dashboards, or computer and smartphone applications. These tools can provide information on the current status of marine ecosystems and the future threat of climate change (Melbourne-Thomas et al. 2021; Trebilco et al. 2021, this issue) and economic activities (Novaglio et al. 2021, this issue) to these systems. They can provide information about ecological outcomes of government policies and link consumers to supply chains and sustainability information on products (Farmery et al. 2021, this issue), and ultimately provide steps that individuals can implement to contribute to positive outcomes for marine environments. Increased uptake and positive outcomes are more likely if the information is locally specific and place-based.

### Overcoming barriers to integrated, ecosystem-based management

As identified in our drivers of change for conservation of biodiversity and ecosystems, movement towards integrated, ecosystem-based management (EBM) will be a key factor in working towards a more sustainable future. Implementing EBM and ecosystem-based fisheries management (EBFM) has been a goal in international environmental laws – implicitly since the 1980s and, more recently, explicitly in legal instruments such as fisheries management agreements and in principles and guidance developed under the Convention for Biological Diversity (Enright and Boteler 2020). However, there remain significant challenges for its effective implementation through formal legal instruments, including the need for co-operation between agencies and more practical guidance about its implementation in different regions and at different governance scales, and the fundamental need for greater political willpower (Enright and Boteler 2020; Rudd et al. 2018). There have been calls for ecosystem approaches that integrate across multiple sectors, and for expanding the concepts of integrated coastal zone management (Post and Lundin 1996) to open ocean systems. Stephenson et al. (2019) describe a pathway towards integrated management for marine systems, identify steps for implementation and consider factors that might enable or inhibit progress towards integrated management. A detailed treatment of actions to progress the successful implementation of integrated, ecosystem-based management is beyond the scope of our study (although many of the actions we identify in Tables 2, 3, 4, 5 could help address this challenge, and build on what is described by Stephenson et al. 2019). Important barriers to achieving integrated EBM and EBFM more broadly are:

- Increased need for understanding of the cumulative effects of the pressures caused by the activities of multiple sectors across multiple jurisdictions (current knowledge gaps are also a consequence of the limited implementation of EBM)
- That adaptive management, while crucial to effective EBM approaches, remains controversial, difficult to implement and enforce, and absent from, or afforded mere lip-service in, most existing legal and policy frameworks (e.g. Enright and Boteler 2020).
- A lack of indicators and reference levels to measure achievements towards EB(F)M, limiting the capacity to implement effective adaptive management approaches
- Limitations in our understanding about the social dimensions of EBM (which encompasses socio-economic-ecological dimensions), particularly in the coastal zone (Le Tissier 2020)
- Lack of tools that consider all dimensions and dynamics, but are efficient and accessible.
- Since EBM is most often system-specific, EBM frameworks need to be tailored to fit the specific context of different systems.
- Limited experience in coordinated planning across agencies and jurisdictions – a task that is fundamental to EBM. In particular, EBM planning involves: (1) cross-jurisdictional engagement for natural systems that cross State and Continental boundaries, and (2) integration of management activities between conservation and resource extraction agencies.

Overcoming these barriers requires secure funding and support for the managers at all levels, to learn and implement ecosystem-based approaches, and could include use of novel technology for testing and monitoring outcomes of management decisions (Fulton 2021). Engagement of stakeholders with ecosystem-based management process is also fundamental, and can be enhanced by employing knowledge brokers and graphic artists who facilitate communication between different disciplines and stakeholders, and working with psychologists to understand biases that may create barriers to participation (Fulton 2021; Stephenson et al. 2019). Finally, clarifying systems and processes for monitoring and responding to changes in marine ecosystems (e.g. through information transfer, as discussed in the section above) could enable adaptive management requirements to be formalized in legal and policy frameworks.

### Shifting towards a more equitable, circular economy

Changing the economic model of profit at the cost of marine ecosystems is critical for marine conservation in the long term. Capitalism has enabled the situation where businesses profit through disproportionately impacting marine ecosystems, but the consequent loss of ecosystem services is felt by all. For example, fewer than 100 companies are responsible for half of the global decline in surface ocean pH to 2015 and 42–50% of increase in mean surface warming to 2010 (Ekwurzel et al. 2017; Licker et al. 2019). Escaping the heavy hand of capitalist interests will require strong governance and, ultimately, social pressure for stronger regulation and more equitable economic markets and sustainability (see also Novaglio et al. 2021; Virdin et al. 2021). It is beyond the scope of this paper to discuss in detail how to change the economic model, however many of our recommended actions could contribute to such a shift. This includes accounting for the economic value of ecosystem goods and services in decision-making processes and increased accountability and transparency around taxation and subsidisation of organisations that pollute or otherwise harm marine ecosystems and development of indicators to support those. While these actions are not sufficient to change the economic model, they are critical steps for safeguarding marine ecosystems into the future.

### Human–environment interactions and COVID

The recent evolution of the COVID-19 global pandemic has changed the course of the next decade and has affected some of the aspects discussed in this paper. For instance, in some countries, a shift in the allocation of funding to new priorities (e.g. medical therapies and research) might delay progress towards meeting some of the UN SDGs (Bates et al. 2020). In addition, reduced food supply during the lockdown in some regions may have elicited illegal fishing (e.g. rural India, Pinder et al. 2020), and reduced control of invasive alien species may have resulted in these species expanding their range (evidence from land, Manenti et al. 2020), with important consequences on biodiversity. While we recognise the disruptive effects of COVID-19 on individuals, society and the environment, we also believe that the pandemic has prompted some positive changes. For example, it has led society to reconsider values and priorities and to discuss alternative economic models that would result in improved societal and environmental outcomes (Cohen 2020). Most importantly, COVID-19 has highlighted the strong link between humans and nature and has demonstrated that large-scale societal changes have the potential to reduce human impacts and benefit biodiversity conservation (Bates et al. 2020). Such benefits include, for example, cleaner air and cleaner and quieter water (Thomson and Barclay 2020), and increased breeding success for some threatened species due to reduced exploitation during lockdown (Bates et al. 2020; Manenti et al. 2020). Regardless of the negative or positive nature of its consequences, COVID-19 has created momentum to catalyse societal consent and undertake actions that will place us on a trajectory towards a more sustainable future. Capitalising on this ephemeral momentum is an opportunity we cannot afford to miss.

## Conclusions

Our global dependence on marine resources and ecosystem services has resulted in the severe degradation of many systems. These impacts are exacerbated by climate change, which is now the long-term driver with the greatest impact on marine ecosystems and biodiversity. However, there are still many opportunities to mitigate cumulative, more immediate impacts in our oceans, with the critical need to protect and maintain biodiversity and ecosystem function broadly recognised. Conservation programs tend to fail because they do not consider social dimensions of conservation (Bennett et al. 2017). These human elements need to be a core focus for improving conservation success, but the question is how to do ‘human-centred’ conservation in a way that ultimately still prioritises biodiversity and ecosystems. This paper is a step in that direction.

We highlight four key drivers of change: financial mechanisms; sectoral stewardship; management and governance; and social impetus for safeguarding marine ecosystems. Importantly, we highlight how considering the interrelationships between these drivers can identify concrete actions for forming a pathway to a more sustainable future. Furthermore, we outline the key factors that determine the capacity for societies to address the drivers.

While individual methods for communication of up-to-date information pertinent to conservation of biodiversity and ecosystems, such as environmental dashboards, or computer and smartphone applications, currently exist and their use is expanding, centralised communication frameworks that act as synapses linking multiple systems and communities across the globe remain aspirational. Such global communication systems would further enhance the clear approach outlined in this paper of incorporating local awareness and knowledge into providing solutions to global scale problems. We highlight how this localised approach allows global issues to be tackled at more tractable scales that create a feeling that change is indeed achievable.

We have articulated an optimistic, sustainable future for global oceans with respect to the conservation of marine biodiversity and ecosystems and importantly, we have outlined how such a future is technically feasible by 2030. This future would go a long way to achieving the UN SDG 14 ‘Life Below Water’ Target 14.2 ‘Protect and Restore Ecosystems’. It should be noted, however, that this target has one indicator: The proportion of national exclusive economic zones managed using ecosystem-based approaches. As over fifty percent of the world’s oceans constitute the high seas (FAO 2020), which are not addressed within SDG 14.2, we purport that in order to more fully achieve a sustainable future for global oceans, mechanisms to develop dynamic ecosystem-based management in the high seas must be included in this future.

## Data Availability

Not applicable.
